# Fate of *Salmonella enterica* Typhimurium and *Listeria monocytogenes* in Black Soldier Fly (*Hermetia illucens*) Larvae Reared on Two Artificial Diets

**DOI:** 10.3390/foods11152208

**Published:** 2022-07-25

**Authors:** Annalisa Grisendi, Francesco Defilippo, Chiara Lucchetti, Valeria Listorti, Matteo Ottoboni, Michele Dottori, Andrea Serraino, Luciano Pinotti, Paolo Bonilauri

**Affiliations:** 1 Istituto Zooprofilattico Sperimentale della Lombardia e Emilia Romagna, Via Pitagora 2, 42124 Reggio Emilia, Italy; chiara.lucchetti@izsler.it (C.L.); michele.dottori@izsler.it (M.D.); paolo.bonilauri@izsler.it (P.B.); 2 Istituto Zooprofilattico Sperimentale della Lombardia e Emilia Romagna, Via Bianchi 9, 24124 Brescia, Italy; francesco.defilippo@izsler.it; 3 Istituto Zooprofilattico Sperimentale del Piemonte, Liguria e Valle D’Aosta, Via Bologna 148, 11012 Torino, Italy; valeria.listorti@izsto.it; 4 Department of Health, Animal Science and Food Safety, VESPA, University of Milan, Via dell’Univerità 6, 20100 Lodi, Italy; matteo.ottoboni@unimi.it (M.O.); luciano.pinotti@unimi.it (L.P.); 5 Department of Veterinary Medical Sciences, University of Bologna, Via Tolara di Sopra 50, 40126 Ozzano dell’Emilia, Italy; andrea.serraino@unibo.it

**Keywords:** novel food, insect, black soldier fly, *Salmonella* typhimurium, *L. monocytogenes*

## Abstract

Ensuring food security is one of the main challenges facing the world over the next 30 years. There is, thus, an urgent need to significantly increase the supply of sustainable protein that can be transformed into animal feed. Proteins from insects offer a valuable alternative. This article presents the results of challenge tests conducted to investigate the dynamics of the microbial load of *Salmonella enterica* Typhimurium and *Listeria monocytogenes* in black soldier fly (*Hermetia illucens*) larvae grown on contaminated substrates. Four separate challenge tests were performed on two substrates: the Gainesville diet and a homemade diet. The challenge test procedure was carried out in accordance with ISO/DIS 20976-2 (*under development*). The results of this study show that, when grown on contaminated substrates, BSF larvae do not eliminate *Salmonella* Typhimurium or *L. monocytogenes*, but can reduce their microbial load. Sanitation processes downstream of the breeding of BSF larvae are, however, required to reduce the microbiological risks of this novel food.

## 1. Introduction

Interest in the human consumption of edible insects (entomophagy) in Western countries is increasing and more and more insect-based food products are being marketed [1]. Since January 2018, insects have been considered as a novel food in Europe, as stated in EU regulations 2015/2283 [2]. The use of insects in both feed and food formulations is increasingly being recognized as a novel way to improve feed and food security [3,4,5]. Edible insects have a high content of protein, essential amino acids, minerals, and vitamins [6,7,8,9]. Their final larval stage (prepupae) has a high content of protein and fat. Prepupae can contain up to 47% of crude protein and 35% of ether extract (on a dry matter basis), depending on the rearing substrate [10,11,12,13,14].

Insects can, thus, be used as either food or feed source and, thus, contribute to solving global food problems [15]. Compared to standard farming, insect rearing requires less energy and very little land. It could potentially result in a lower environmental footprint and, thus, have a beneficial effect on the environment [15,16,17].

To support safety evaluations and risk assessments, as well as to establish specific microbial criteria for edible insects by the European Food Safety Authority (EFSA), additional quantitative data on their microbial quality are needed [18,19]. As highlighted by the EFSA, microbiological data are scarce [20]. In fact, few studies contain data on the intrinsic properties of pH and water activity (a_w_) in the diet, despite them having an important impact on the growth and survival of pathogenic microorganisms. These factors, thus, need to be taken into account when considering insects as either feed or as part of the food matrix [21].

Farmed insects are considered by the current EU regulation as ‘farmed animals’ and, thus, they have to be fed using safe substrates [22]. Reared insects have to be safe not only for animals, but also for human consumption; however, like any other farmed animals, the substrates (feed) used to grow insects can be contaminated by pathogens [23].

Previous studies reported that black soldier flies (BSFs) act on microbial contamination by modifying it in the substrate and in the larvae [24,25,26,27]. However, currently, there are no microbiological challenge tests available to verify the fate of foodborne pathogens in larvae grown on a substrate contaminated from the egg to the prepupal stage.

This study aims to assess the level of contamination of BSF larvae grown on a substrate highly contaminated by foodborne pathogens in order to give useful results for a risk assessment conducted to evaluate the food and feed safety if a substrate accidentally contaminated by pathogens is used in the breeding of this important source of protein for food and feed.

In the present study, two substrates were contaminated with either *Salmonella enterica* serotype Typhimurium or *Listeria monocytogenes* in two independent challenge tests and a reduction in pathogen load in the larvae and substrate was investigated. The results of this study provide useful information aimed at improving *H. illucens* rearing practices. In addition, the results could be used in future microbiological risk assessments on BSF larvae.

## 2. Materials and Methods

### 2.1. Rearing Conditions of H. illucens

An *H. illucens* stable laboratory colony was established at IZSLER in Reggio Emilia (Italy) starting with 200 larvae and 200 pupae provided by the University of Milan (Italy). The rearing was conducted in climatic chambers under controlled conditions (25 °C ± 1 °C, 70% ± 5% relative humidity (RH), and 16:8 light: dark photoperiod for adults and darkness for pupae and larvae). Larvae were placed in separate plastic 40 × 20 × 20 cm boxes with the Gainesville diet, until the pupal stage was reached. Pupae were placed in a mating and oviposition 50 × 50 × 170 cm box until the adult stage. Adults were fed with a saturated solution of water. A cage (15 × 10 × 10) with the Gainesville diet and slices of corrugated carboard was placed in the oviposition box, as a support for oviposition. The corrugated cardboard was checked daily. When eggs were found, the cardboard was placed into a glass jar which was maintained in darkness for 1 or 2 days in order to hatch. Young larvae were counted and approximately 250–300 of them were allocated to the selected diet.

Larvae were fed with two different substrates: either the Gainesville diet (G) or a homemade artificial diet (D). Diet G (50% wheat bran, 30% alfalfa meal, 20% corn meal) was developed for rearing house flies and it is currently recommended for mass rearing of BSFs [27,28]. Diet D is an artificial diet consisting of agar (5 g), lentil flour (4 g), wheat bran (10 g), corn flour (7 g), wheat germ (3 g) and water (80 mL). A larval growth study was carried out for both substrates.

Three BSF life cycles were completed on each substrate and data were collected from each larval generation. The developmental time of the larvae and pupae, percentage of adult emergence and sex ratio were measured and then used to compare the growth efficiency of the two diets.

The feed conversion ratio (FCR), which is the amount of feed needed (in kg) to obtain one kg of weight increase in the larvae, was not calculated in this study. The FCR data reported in the literature [29,30,31] were used to discuss the theoretical contribution of FCR to the results obtained.

### 2.2. Pathogen Dynamics in BSF and Two Growing Substrates

#### 2.2.1. *Salmonella* Typhimurium and *Listeria monocytogenes* Challenge Tests

To rule out any effect of the type of rearing substrate on the larval behavior, this study was carried out on two different substrates: a standard Gainesville diet and an alternative homemade diet. The fate of *Salmonella enterica* Typhimurium and *Listeria monocytogenes* in the BSFs as well as in each of the two diets was investigated in four separate challenge tests.

Eight 40 × 40 × 20 cm boxes with 100 g of substrate for each of the two diets were used in each experiment. The substrate was initially placed in a corner of the box to maintain its humidity. Three boxes were used as control samples and, therefore, no BSF eggs were placed onto the substrate. BSF larvae were placed in the remaining five boxes. All boxes were then incubated at 25 °C and a relative humidity of 70 ± 5% in dark conditions.

Before the larvae were added, each diet was artificially contaminated with 2 mL (surface sprayed) of a cocktail containing three strains of *Salmonella enterica* serotype Typhimurium: a strain of collection ATCC^®^14028 and two strains of the same serotype isolated from pork meat (natural contamination). Again, for the *L. monocytogenes* challenge test, a cocktail of three strains was used: one strain of collection ATCC^®^13932 and two strains isolated from pork meat (natural contamination).

All *Salmonella* Typhimurium and *L. monocytogenes* strains were first cultured in nutrient broth (Oxoid AB, Malmö, Sweden) for at least 12 h at 37 °C and then in order to adapt the strain to the challenge test conditions, the broth was kept at 25 °C for 3 days. After adaptation, the three strains were mixed well and inoculated into the two diets. The inoculums reached an early stationary phase of about 1 × 10^8^ CFU/mL by the McFarland comparison scale, for both *Salmonella enterica* Typhimurium and *L. monocytogenes* experiments. The challenge test procedure was carried out in accordance with ISO/DIS 20976-2 (*under development*).

Larvae were observed daily and their stage of development was determined. Starting on the day of contamination, for a total of five times, according to the BSF stage change, a sample of 10 g of rearing substrate was provided. From day 3, a sample of ten larvae, according to the development stage, was collected for microbiological enumeration of pathogens. Due to the fact that the weight of a larva varies during the development and at days 1 and 2 after hatching, larvae are very light (0.01 g dry weight at day 2). Consequently, 1- or 2-day-old larvae were not analyzed. The weight of the larvae used in each sampling point was always diluted 1:10 for microbiological quantification. Prior to these analyses, larvae samples were first washed once using ethanol 70% *v*/*v* and then three more times using pure water.

*Salmonella* spp. was detected according to ISO 6579-1:2017. Briefly, since ISO 6579-1:2017 is validated for all sample sizes lower than 25 g, 1 to 5 g of insects was enriched (1:10) in buffered peptone water for 18 ± 2 h at 36 °C ± 2 °C and 100 µL of sample was distributed on an MSRV agar plate. Plates were incubated for 24/48 h at 41.5 °C ± 1 °C. The presence of *S.* Typhimurium was confirmed using Xylose-Lysine-Desoxycholate (XLD) Agar and appropriate biochemical miniaturized tests (Microgen GnA+B-ID system—Microgen Bioproducts Ltd., Camberley, UK).

*Salmonella* Typhimurium was counted by Micro MPN enumeration according to part 2 of ISO 6579:2012. When the load of pathogens was above 1000 CFU/g, the 1:10 initial sample dilution was additionally diluted 10-fold and then directly plated on XLD and Hektoen enteric agar, with a detection limit of 10 CFU/g.

*L. monocytogenes* was detected and counted according to ISO 11290-1:2017 and ISO 11290-2:2017. Briefly, since the two aforementioned ISOs are validated for all sample sizes lower than 25 g, 1 to 5 g of insects was enriched in Half Fraser Broth and plated in Agar *Listeria* according to Ottaviani and Agosti (ALOA) and OXFORD agar. Enumeration was performed by 10-fold dilution and direct plating on ALOA agar with a detection limit of 10 CFU/g. All counts were repeated in triplicate and data presented as the mean of the three replicates. The same procedure was also used for the substrate (both treated and untreated) analysis.

#### 2.2.2. Physicochemical Analyses

The water activity (a_w_) and pH were analyzed in the substrates (G and D diets) contaminated with *Salmonella* Typhimurium with and without larvae (controls) at days 1–2–6–8–10–13 and 23. The a_w_ was analyzed following ISO 18787:2017.

The pH was determined according to the MFHPB—03:2014 method for the determination of pH of foods, using the AQUALAB 4TE machine (METER group).

### 2.3. Statistical Analysis

The larval and pupal length and weight, which were collected per experimental replicate, were then reported as mean ± standard deviation. Larval length, weight as well as pupal weight were compared between the two diets (G and D) by one-way ANOVA.

Pathogen enumerations were transformed to decimal log and a non-thermal inactivation curve for vegetative microorganisms was drawn. The data were subjected to one-way ANOVA with Bonferroni post hoc tests to test significant differences in the load of *Salmonella* Typhimurium and *L. monocytogenes* Log (CFU/g) (dependent variables) between time, diets (the two independent challenge tests), substrates on which larvae were grown, larvae alone and controls (substrates alone) and their interactions (independent variables). All analyses were conducted with STATA 7.0, College Station, TX, USA. In all the statistical comparisons, *p* < 0.05 was selected as the significance threshold.

## 3. Results

### 3.1. Pathogen Dynamics in BSF and the Growing Substrates

To determine whether the two proposed diets (D and G) were equally suited to support growth, BSF growth trends as well as all biological parameters were collected and compared. The two diets showed similar results and were both found to be suitable for BSF rearing (see Supplementary Materials, Table S1).

### 3.2. Salmonella enterica Typhimurium Challenge Test

The progress of *Salmonella enterica* Typhimurium contamination (Log CFU /g) of substrates, larvae and control groups (substrate without larvae) for each of the two challenge tests performed was monitored over a 17-day period. All *Salmonella enterica* Typhimurium concentrations measured are reported in Table 1.

**Figure 1:**
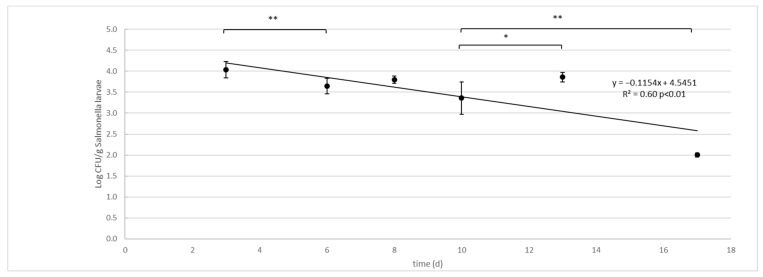
*Salmonella enterica* Typhimurium decay in larvae (Log CFU/g) of black soldier fly (*H. illucens*) reared on artificial contaminated substrate. Vertical bars represent standard deviation of 6 rep. in two independent challenge tests. Horizontal bars show significant differences among experimental points, * *p* < 0.05; ** *p* < 0.01. The equations for the regression line and R2 are presented together with the *p* significant of the model.

The ANOVA test showed significant differences (*p* < 0.01) in *Salmonella* concentrations between time (d) and groups (substrates were larvae grown, larvae and substrate without larvae, ctrl) and their interactions (time * groups). No differences were observed between diets. Due to the lack of differences in the results between the two diets, data from the two independent challenge tests were merged and the mean and standard deviation were recalculated and used to model the survival curve (Table 2, Figure 1). The *Salmonella* contamination of substrate, larvae and controls decreased significantly during the larval development time, with significant differences between groups (Table 2). Comparing the contamination between the substrate with larvae and the control without larvae, the control substrate always had a significantly (*p* < 0.01) higher concentration of *Salmonella,* except on day 13. The *Salmonella* concentration in the larvae was significantly lower than that of their substrate on day 3 and on the last two experimental dates (13 and 17 days). On the other hand, in the period between, the larvae showed the same concentration as their growth substrate. The *Salmonella* concentration significantly decreased over time in all three experimental groups. The fastest reduction was observed in the larvae (D = 6.88 logCFU/g between day 3 and day 17), compared to their substrate (D = 18.58 log CFU/g between day 3 and day 17). In the Control group, the decay of *Salmonella* was extremely low in the first 6 days (D = 68.94 logCFU/g) and, subsequently, accelerated between day 8 and day 17 (D = 9.86 logCFU/g).

The survival curve presents as a linear trend with a constant rate of *Salmonella* decay from day 3 to the end of the experiment (Figure 1):

$$\log CFU/g=-0.1154x+4.5451\;\left(3<x<17days\right)-R2=0.60\;p<0.01$$

### 3.3. Listeria monocytogenes Challenge Test

The fate of *L. monocytogenes* contamination of substrates, larvae and control groups (substrate without larvae) for each of the two diets was monitored over a 17-day period. All *L. monocytogenes* concentrations counted in Log CFU /g are reported in Table 3.

With regard to the *Salmonella enterica* Typhimurium challenge test, due to extreme lightness of the larvae on days 1 and 2, the counts of *L. monocytogenes* started on day 3.

Due to their light weight, 1- and 2-day-old larvae were excluded from the microbiological analysis.

**Figure 2:**
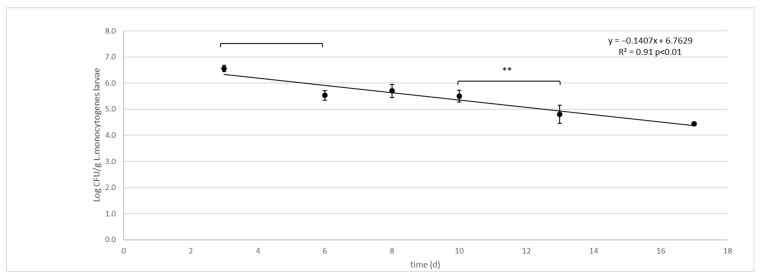
*L. monocytogenes* decay in larvae (Log CFU/g) of black soldier fly (*H. illucens*) reared on artificial contaminated substrate. Vertical bars represent the standard deviation of 6 rep. in two independent challenge tests. Horizontal bars show significant differences among experimental points, ** *p* < 0.01. The Equation for the regression line and R2 are presented together with the *p* significant of the model.

Further, in the *L. monocytogenes* challenge test, the ANOVA test showed significant differences (*p* <0.01) in the *L. monocytogenes* concentration between time(d) and groups (substrates were larvae grown, larvae and substrate without larvae, ctrl) and their interactions (time*groups). No differences were observed between diets. Similar to the *Salmonella enterica* Typhimurium experiments, due to the lack of differences in the results between the two diets, data from the two independent challenge tests were merged and the mean and standard deviation recalculated and used to model the survival curve (Table 4, Figure 2). *L. monocytogenes* contamination of substrate, larvae and controls decreased significantly during larval development time, with significant differences between groups (Table 4).

Comparing the contamination between the substrate with larvae and the control without larvae, the control substrate always showed a significantly (*p* <0.01) higher concentration of *L. monocytogenes*. Starting from day 6, the *L. monocytogenes* concentration in the larvae was significantly lower than that of their substrate; the larvae showed the same concentration as their growth substrate only on day 3 (first day of the microbiological quantification). The *L. monocytogenes* concentration significantly decreased over time in larvae and their growing substrate, but not in the control group (inoculated substrate without larvae). The fastest reduction was observed in the larvae (D = 6.61 logCFU/g between day 3 and day 17), compared to their substrate (D = 21.45 log CFU/g between days 3 and 17). In the Control group, the decay of *L. monocytogenes* was extremely low throughout the experimental time (D = 57.56 logCFU/g).

The survival curve presents as a linear trend with a constant rate of *L. monocytogenes* decay from day 3 to the end of the experiment (Figure 2):

$$\log CFU/g=-0.1407x+6.7629\;\left(3<x<17days\right)\;R2=0.91\;p<0.01$$

### 3.4. Comparison of Salmonella enterica Typhimurium and L. monocytogenes Challenge Test

*Salmonella enterica* Typhimurium contamination was significantly lower (*p* < 0.01) than *L. monocytogenes*, in both the substrate with the larvae and in the controls without larvae, but only from day 2. No significant differences were observed on day 1 after inoculums. From day 3, the first day of microbiological enumeration of pathogens in larvae, the *L. monocytogenes* load was always significantly higher than for *Salmonella enterica* Typhimurium (Table 5, *p* < 0.01).

### 3.5. Physicochemical Analyses

The water activity a_w_ and pH of the substrates were measured and their dynamics analyzed (Table 6). Data from the *Salmonella enterica* Typhimurium and *L. monocyotgens* experiments were merged and larvae values represent the mean and standard deviation of their growing substrate. In the controls (contaminated substrate without larvae), the pH remained in the range of normality (6.59–8.08) in both diets until the end of the experiments, showing a slow increment toward basic values during the experiments. Larval activity seemed to influence the pH trend of the diets during growth, in particular, for diet D, where larvae induced a significantly higher pH for the substrate than the control (*p* < 0.05). The a_w_ values in the controls remained stable only until day 10, after which the values decreased, reaching 0.65 at the end of the experiment, probably due to air drying of the substrates. When larvae were present, a_w_ values remained stable throughout the study.

## 4. Discussion

It is now internationally accepted by scientists and economists that the world population will increase to 9.6 billion by 2050. Current food production cannot support such a large number of people and it is estimated that this will need to almost double [32]. Insects can be an important alternative source of food production for humans and farmed animals, and using insects for feed and food is an attractive option. Insects have high nutritional value, a high reproduction rate and high feed conversion efficiency, thus, making them ideal for agricultural purposes. In addition, compared to other farmed animals, insects seem to be more “respectful” of the environment [29,30].

Based on current legislation, animals in the EU must be fed only with safe feed (Commission Regulation (EC) No. 68/20136, Regulation (EC) No. 178/2002, and Regulation (EC) No. 767/2009), and the Regulation (EC) No. 1069/20097, which considers insects as “farmed animals”, does not allow the use of certain substrates (e.g., manure, catering waste or former foodstuffs containing meat and fish) for their feeding [20].

The black soldier fly (BSF), *Hermetia illucens*, Diptera of the family Stratiomyidae, thrives on an immense variety of organic substrates, which is why it is suitable for small-scale waste management purposes using substrates, such as manure [33]. These biological characteristics make the species an ideal candidate to support a market based on the circular economy, as described in [34].

This study involves the first challenge tests to study the inactivation potential and kinetic parameters of BSF larvae reared on substrates contaminated with *Salmonella enterica* Typhimurium and *Listeria monocytogenes*. In addition, for what we believe is the first time, results are reported here on *L. monocytogenes*, which provide important information for risk managers in view of the ubiquity of this telluric bacterium in all environments.

One limitation of this study is that larvae at 2 days of age weigh approximately 0.01 g (dry weight) and one day after hatching, they weigh approximately half this, thus, making microbiological analysis difficult. For this reason, the concentration of pathogens was only measured starting on day three after hatching. However, given that the stage of development of commercial interest is that of the prepupa, which is reached, in our conditions of study, after about 17 days, this limitation seems acceptable.

Another limitation is that our experimental plan was aimed at studying the inactivation capacity of a process against a foodborne pathogen (ISO 20976-2: challenge tests to study inactivation potential and kinetic parameters; draft under development), without demonstrating the possibility that low contamination of the substrate could magnify itself in the larvae during the production period (ISO 20976-1:2019: challenge tests to study growth potential, lag time and maximum growth rate).

Despite this limitation, the results of this study show that the BSF can be reared on a substrate contaminated with *Salmonella* and *L. monocytogenes*, obtaining a reduction in the microbial load of both pathogens. However, the reduction observed was incomplete. The slow rate of pathogen decay, observed both in *Salmonella* and *L. monocytogenes* experiments, needs to be carefully evaluated. In fact, it could represent a source of concern regarding the potential presence of a subpopulation of more resistant (persistent) bacteria. Moreover, the reduction rate observed in both pathogens in comparison to larval developmental time (D of about 7 days, measured between 3- and 17-days Vs 20 days mean developmental time) will allow a maximum reduction of about two decimal points in the pathogen load during the farming time.

Erickson et al. [24] evaluated the effects of BSF larvae on cow, hog, or chicken manure inoculated with 10^7^ CFU/g of *Salmonella* and *E. coli* O157:H7. They obtained a greater reduction in the contamination of both pathogens than in the control inoculated without the larvae, but starting with larvae already grown (10–11 days old). Lalander et al. [25] observed a strong reduction in the concentration of *Salmonella* in organic waste (pig manure mixed with dog food) in a continuous flow fly larvae reactor, but did not evaluate the contamination of the larvae.

These literature results, even with the differences underlined, can be considered as in agreement with our findings for *Salmonella*. Regarding *L. monocytogenes* experiments, the microbial load from day 3 until the end of the experiment was different from that of *Salmonella*. *L. monocyotogenes*, which was significantly more concentrated, although the load of pathogens in the growth substrates on day 1 was not different, with one log difference on day 13 and about two log at the end of the experiments (Table 5).

This difference in the pathogen dynamics may reflect the diversity of their bacterial cell walls and, in general, the intrinsic greater resistance to environmental stresses of *L. monocytogenes* compared to *Salmonella*. A study conducted by Choi et al. [35] observed antimicrobial activity of larval extracts on Gram-negative bacteria, but no or very little on Gram-positive bacteria. These antimicrobial agents derived from the larvae may be among the substances that are produced in the larval body for their survival and, thus, they may have an antimicrobial action in the gut of larvae when pathogens are ingested.

On the other hand, the results reported by Park et al. [24] seem to confirm that, when induced by a septic needle, *H. illucens* larvae produce an immune response that has a greater antibacterial activity, even against Gram-positive bacteria, such as methicillin-resistant *Staphylococcus aureus*. The larvae in our study came into contact with a high concentration of *L. monocytogenes* after hatching. It is, thus, possible that a pathogen infection in the larvae may have actually stimulated an immune response against the pathogen with a similar mechanism.

The pathogens were present both in the substrate in which the larvae grew and in the control without larvae (Table 2 and Table 4). The effect on the substrate can always be discussed assuming a direct action of the larvae, as also previously reported [25,26]. However, it is worth noting that the decay of pathogens was significantly faster in the larvae than in the growth substrate. This is even more relevant considering a feed conversion ratio for the larvae of between 1.4 and 2.6, in relation to the quantity of proteins and fats contained in the diet of BSF larvae [31]. In theory, the concentration should be approximately twice as high in the larvae as in the substrate, providing the larvae had a direct action on the decay of pathogens, but several, not completely known, factors may influence the contamination of larvae grown on a substrate contaminated by foodborne pathogens. On the whole, the results of this study show that larvae grown on a substrate contaminated by foodborne pathogens have a lower pathogen load than the substrate; the quantification of this difference allows one to predict the final contamination of larvae depending on the initial contamination of the substrate, therefore, suggesting the possibility of classifying the different growth substrates to be used to breed BSF larvae for safety implications [36,37], making this breeding more attractive to the market.

However, the pathogens also decayed in the control where no larvae were added. This decay is attributable to a decrease in water activity, which, after 10 days, fell below 0.95 and then fell below approximately 0.70 at the end of the experiment (Table 6). This can lead to stress and, therefore, a reduction in the number of microorganisms for both pathogens. However, this reduction was slower than that observed in the larvae, both for *Salmonella* and especially for *L. monocytogenes*, which again, demonstrated a greater resistance to environmental stresses on this occasion.

The pH remained fairly constant around neutral values in the controls, while in the D diet, the action exerted by the larvae involved an increase of about 1 pH point compared to the G diet (Table 6). It has been hypothesized that the antimicrobial compounds present in *H. illucens* larvae are either less stable or less active at lower pH values [38]. In our experiments, the differences in pH of the two tested diets did not seem to interfere with the ability of larvae to reduce the pathogen load. In fact, the diet was never found to be a significant parameter in the ANOVA test in the two challenge tests (*p*> 0.05).

## 5. Conclusions

Insects in current European legislation fall within the category of “novel food ingredients”, and, therefore, for them to be placed on the market, microbiological food safety concerns need to be addressed. A study conducted in Belgium found that the microbiological load of untreated insects always exceeds the food safety criteria for fresh minced meat [37] and blanching treatment manages to reduce this contamination in different ways between species and treatments (time–temperature dependent) without, however, eliminating it [39,40]. The average reduction obtained after blanching treatment in the total aerobic count was around 3 to 4 logCFU/g, depending on insect species, while the total microbial load of fresh insects was 7–8 log CFU/g [31].

The results of our study confirm that BSF larvae can grow in contaminated substrates, thus, reducing *Salmonella* and *L. monocytogenes* contamination through their direct action on the growth substrate. This is particularly evident in comparison with controls, where *Salmonella* and *L. monocytogenes* contamination decreased at a significantly slower rate (*p* < 0.01). As long as the contamination level is reasonably low, the BSF larvae could, thus, be bred on contaminated substrates. In any case, we confirmed that at the end of the farming period, when larvae grow on a highly contaminated substrate, the results were also contaminated. As reported in the study by Lalander et al., which focused exclusively on *Salmonella* [25], this underlines the importance of post-production treatment before their use as food or feed.

The feeding activity of larvae modified the physical (pH and a_w_) and microbiological characteristics of the substrate, and this modification could be influenced by the diet’s components. In the present study, during their developmental stage, the larvae reduced the concentration of *Salmonella* and *L. monocytogenes* faster than the natural decay of pathogens observed in the controls.

Considering these observations and the results of this study, a risk assessment study would be possible to evaluate different possible growth substrates at different levels of microbial contamination. The aim would be to calculate the probability that residual contamination by *Salmonella* and *L. monocytogenes* can remain in the larvae after farming and blanching treatment.

The antimicrobial properties of larvae are not yet completely understood; however, at least against *Salmonella*, our results confirm the interesting abilities of BSF larvae that could be exploited for food and feed production. If, following a rigorous risk assessment, also based on the results of this study, the possibility of using substrates potentially contaminated by *Salmonella* and/or *Listeria monocytogenes* should emerge, without this entailing unacceptable risks for the consumer (even clearly following post-production treatments aimed at further improving safety), this would make this breeding more attractive in a market that must increasingly turn towards a circular economy.

## Figures and Tables

**Table 1:** *Salmonella enterica* Typhimurium concentrations (Log CFU/g, and standard deviation SD) in substrates, larvae and control groups (substrate without larvae) for each of the two diets over a 17-day period. Due to their light weight, one- and two-day-old larvae were excluded from the microbiological analysis.

	Diet D—Log CFU/g (SD)			Diet G—Log CFU/g (SD)		
Days	Substrate	Larvae	CTRL	Substrate	Larvae	CTRL
1	7.26 (0.05)		7.48 (0.02)	7.32 (0.05)		7.91 (0.00)
2	6.83 (0.02)		7.45 (0.01)	6.86 (0.03)		7.74 (0.01)
3	4.34 (0.09)	4.00 (0.21)	7.30 (0.07)	4.64 (0.21)	4.08 (0.23)	7.32 (0.04)
6	3.86 (0.14)	3.64 (0.22)	7.60 (0.05)	3.49 (0.20)	3.64 (0.19)	7.32 (0.08)
8	3.65 (0.11)	3.80 (0.09)	5.30 (0.04)	3.79 (0.10)	3.79 (0.10)	4.99 (0.09)
10	3.65 (0.15)	3.04 (0.16)	5.32 (0.04)	3.42 (0.10)	3.68 (0.21)	5.78 (0.04)
13	4.81 (0.11)	3.80 (0.10)	4.70 (0.01)	4.65 (0.12)	3.92 (0.11)	5.95 (0.05)
17	3.78 (0.06)	2.00 (0.07)	4.30 (0.02)	3.70 (0.04)	2.00 (0.04)	4.17 (0.09)

**Table 2:** *Salmonella* decay in the substrate, larvae and CTRL (substrate without larvae) Log CFU/g (standard deviation). Data obtained in six replications in two independent challenge tests. The letters ^a, b, c^ indicate differences between groups (substrate, larvae and CTRL) on the same sampling date; the letters ^x, y, z, j, k, w^ indicate differences between dates within the same group. One- and two-day-old larvae (0.01 g each) were excluded from the microbiological analysis.

	Log CFU/g (SD)		
Days	Substrate	Larvae	CTRL
1	7.29 (0.06) ^a,x^		7.69 (0.24) ^c,x^
2	6.84 (0.24) ^a,y^		7.59 (0.16) ^c,x^
3	4.49 (0.22) ^a,z^	4.04 (0.20) ^b,x^	7.31 (0.05) ^c,x^
6	3.67 (0.25) ^a,j^	3.64 (0.18) ^a,y^	7.46 (0.16) ^c,x^
8	3.72 (0.12) ^a,j^	3.80 (0.09) ^a,y^	5.15 (0.18) ^c,y^
10	3.54 (0.17) ^a,j^	3.36 (0.39) ^a,z^	5.55 (0.25) ^c,y^
13	4.73 (0.13) ^a,k^	3.86 (0.11) ^b,y^	5.33 (0.69) ^a,y^
17	3.74 (0.06) ^a,w^	2.00 (0.05) ^b,k^	4.24 (0.09) ^c,z^

**Table 3:** *L. monocytogenes* concentrations (Log CFU/g, and standard deviation SD) in substrates, larvae and in control groups (substrate without larvae) for each of the two diets over a 17-day period. Due to their light weight, one- and two-day-old larvae were excluded from the microbiological analysis.

	Diet D—Log CFU/g (SD)			Diet G—Log CFU/g (SD)		
days	Substrate	Larvae	CTRL	Substrate	Larvae	CTRL
1	7.30 (0.09)		7.72 (0.02)	7.17 (0.10)		7.79 (0.01)
2	6.98 (0.04)		7.48 (0.06)	6.95 (0.05)		7.38 (0.02)
3	6.62 (0.02)	6.65 (0.10)	7.00 (0.09)	6.55 (0.03)	6.48 (0.08)	7.00 (0.04)
6	6.15 (0.06)	5.37 (0.03)	7.84 (0.15)	6.21 (0.07)	5.70 (0.01)	7.40 (0.06)
8	6.74 (0.05)	5.48 (0.06)	7.59 (0.10)	6.40 (0.05)	5.93 (0.05)	7.40 (0.19)
10	6.40 (0.35)	5.70 (0.05)	7.96 (0.08)	6.48 (0.33)	5.30 (0.04)	7.68 (0.15)
13	6.53 (0.04)	4.67 (0.35)	7.08 (0.12)	6.08 (0.07)	4.96 (0.33)	7.79 (0.11)
17	5.96 (0.11)	4.49 (0.04)	7.46 (0.08)	5.91 (0.09)	4.40 (0.03)	7.49 (0.08)

**Table 4:** *L. monocytogenes* decay in the substrate, larvae and CTRL (substrate without larvae) Log CFU/g (standard deviation). Data obtained in 6 rep. in two independent challenge tests. The letters ^a, b, c^ indicate differences between groups (substrate, larvae and CTRL) on the same sampling date. The letters ^x, y^ and ^z^ indicate differences between dates within the same group. One- and two-day-old (0.01 g each) larvae were excluded from the microbiological analysis.

	Log CFU/g (SD)		
Days	Substrate	Larvae	CTRL
1	7.24 (0.11) ^a,x^		7.75 (0.04) ^c,x^
2	6.96 (0.17) ^a,x^		7.43 (0.07) ^c,x^
3	6.59 (0.05) ^a,y^	6.56 (0.12) ^a,x^	7.00 (0.06) ^c,y^
6	6.18 (0.07) ^a,z^	5.53 (0.18) ^b,y^	7.62 (0.26) ^c,x^
8	6.57 (0.19) ^a,y^	5.70 (0.25) ^b,y^	7.49 (0.17) ^c,x^
10	6.44 (0.26) ^a,y^	5.50 (0.22) ^b,y^	7.82 (0.19) ^c,x^
13	6.30 (0.26) ^a,y^	4.81 (0.34) ^b,z^	7.43 (0.40) ^c,x^
17	5.93 (0.09) ^a,z^	4.45 (0.06) ^b,z^	7.48 (0.07) ^c,x^

**Table 5:** *Salmonella enterica* Typhimurium and *L. monocytogenes* decay in larvae Log CFU/g (standard deviation). Data obtained in four independent challenge tests. The letters ^a^ and ^b^ indicate significant *p* < 0.01.

	Log CFU/g (SD) in Larvae	
Days	*Salmonella enterica* Typhimurium	*L. monocytogenes*
3	4.04 (0.20) ^a^	6.56 (0.12) ^b^
6	3.64 (0.18) ^a^	5.53 (0.18) ^b^
8	3.80 (0.09) ^a^	5.70 (0.25) ^b^
10	3.36 (0.39) ^a^	5.50 (0.22) ^b^
13	3.86 (0.11) ^b^	4.81 (0.34) ^b^
17	2.00 (0.05) ^b^	4.45 (0.06) ^b^

**Table 6:** The dynamics of water activity (a_w_) and pH in the two tested substrates (D and G).

	Diet D (SD)				Diet G (SD)			
	a_w_		pH		a_w_		pH	
Days	CTRL	larvae	CTRL	Larvae	CTRL	Larvae	CTRL	Larvae
1	0.980 (0.001)	0.980 (0.001)	6.69 (0.01)	6.87 (0.01)	0.98 (0.02)	0.980 (0.02)	6.59 (0.02)	7.53 (0.01)
2	0.982 (0.002)	0.980 (0.001)	6.58 (0.02)	7.47 (0.02)	0.98 (0.02)	0.943 (0.01)	6.83 (0.02)	6.64 (0.02)
6	0.982 (0.001)	0.970 (0.003)	8.08 (0.01)	8.23 (0.01)	0.98 (0.01)	0.971 (0.01)	7.00 (0.02)	6.88 (0.01)
8	0.971 (0.002)	0.980 (0.002)	7.95 (0.02)	8.52 (0.01)	0.97 (0.01)	0.971 (0.01)	7.78 (0.01)	6.97 (0.02)
10	0.943 (0.002)	0.970 (0.003)	7.76 (0.01)	7.47 (0.01)	0.945 (0.01)	0.972 (0.03)	7.08 (0.02)	7.23 (0.01)
13	0.773 (0.002)	0.970 (0.001)	7.30 (0.02)	8.23 (0.02)	0.85 (0.01)	0.973 (0.01)	7.15 (0.02)	6.64 (0.01)
17	0.661 (0.005)	0.980 (0.002)	6.98 (0.01)	8.35 (0.01)	0.64 (0.01)	0.971 (0.02)	7.18 (0.01)	6.97 (0.01)

## Data Availability

The data presented in this study are available on request from the corresponding author.

## Supplementary Materials

The following supporting information can be downloaded at: https://www.mdpi.com/article/10.3390/foods11152208/s1. Table S1. Biological parameters of *H. illucens* when reared on either Gainesville diet (G) or homemade diet (D) over three generations. The table reports the mean values of replicates ± their standard deviation.
