# Supplementary material for: Fate of Salmonella enterica Typhimurium and Listeria monocytogenes in Black Soldier Fly (Hermetia illucens) Larvae Reared on Two Artificial Diets
Source: Foods. 2022 Jul 25;11(15):2208. doi: 10.3390/foods11152208 (PMC9332512; doi:10.3390/foods11152208)
Supplement: Supplementary file 1 [file foods-11-02208-s001.zip › foods-1802518-supplementary.pdf]

Supplementary

In Table S1 are reported results concerning the biological parameters observed during the rearing of larvae on the two diets (G and D). The pupal stage was achieved after 18d and 21d (for G and D diet respectively) after the egg-hatch. No significant difference ($p>0.05$) was observed in larval and pupal final weights between the two diets and also the grow rate of larvae was not different ($p>0.05$). Nevertheless the larvae need more days on diet D and reach the optimum on 22d. Percentages of adult emergence from the pupae reared on G and D diet remain similar (Table S1).

Table S1. Biological parameters of *H. illucens* reared on Gainesville diet (G) and homemade diet (D) through three generations (mean ± Sd).

Parameters	Diet	Values
Time from eggs to pupa (days)	G	18±0.0
	D	21±1.5
Time from pupa to adult (days)	G	30.7±2.3
	D	35±0.0
Larval weight (g)	G	0.7±0.1
	D	0.5±0.1
Pupal weight (g)	G	0.09±0.01
	D	0.1±0.0
% emergence	G	90.4±2.5
	D	90.3±1.5
